# Dietary Habits and Gaming Behaviors of Portuguese and Brazilian Esports Players

**DOI:** 10.3390/nu15194200

**Published:** 2023-09-28

**Authors:** Fernando J. Ribeiro, Raquel Teixeira, Rui Poínhos

**Affiliations:** 1 Faculty of Nutrition and Food Science, University of Porto (FCNAUP), Rua do Campo Alegre, 823, 4150-180 Porto, Portugal; raquel.teixeira1@hotmail.com (R.T.); ruipoinhos@fcna.up.pt (R.P.); 2 Faculty of Sciences, University of Porto (FCUP), Rua do Campo Alegre, 1021, 4169-007 Porto, Portugal

**Keywords:** esports, video games, nutrition, diet, dietary supplements, caffeine, Mediterranean diet, PREDIMED, gaming disorder, IGDS9

## Abstract

As the esports industry grows, marketing campaigns for fast food, soft drinks, alcoholic and energy drinks, and dietary supplements at related events intensify. Portuguese and Brazilian esports players’ dietary patterns remain unexplored. This study aimed to characterize the dietary and gaming habits of esports players. We applied an anonymous, open online survey to a convenience sample of esports players that included the PREDIMED and the Internet Gaming Disorder Scale. The survey was shared through email and social media platforms, including Facebook, Twitter, Instagram, Discord, WhatsApp, and Twitch. The sample (*n* = 579) was predominantly male (91.4%), averaged 26.1 (SD = 7.0) years, and 25.9 (SD = 5.5) kg/m^2^. Most reported a weak (53.7%) adherence to the Mediterranean diet. Almost a third (32.3%) consumed dietary supplements. Our sample presented a low adhesion to the Mediterranean dietary pattern, low consumption of fruit and vegetables, and high consumption of fast food, red and processed meat, soft drinks, and dietary supplements, including caffeine-based supplements. Dietary supplement consumption was related to a higher adhesion to the Mediterranean diet, and a higher level of professionalization and internet gaming disorder correlated with a lower adhesion. In conclusion, we infer that Portuguese and Brazilian esports players follow an unbalanced diet.

## 1. Introduction

Esports involves the competitive practice of video games against other players. Some of the most well-known esports games include first-person shooters such as Counter-Strike: Global Offensive and multiplayer online battle arena games such as League of Legends [1]. This hypercompetitive activity demands a high level of cognitive aptitude, mental agility, fine motor skills, fast reaction times, and resistance to mental fatigue [2].

The esports industry has been expanding and gaining an audience at an extraordinarily fast pace. The worldwide overall viewership for esports is expected to reach around 600 million people around 2023 [3]. The number of individuals who regularly play video games is also increasing and has possibly reached a staggering value of 2.7 billion in 2020 [4]. The United States, Germany, China, Republic of Korea, and South Africa are some of the countries that have recognized esports as an official sport [5]. They officially featured in the lead-up to the 2018 Olympic Winter Games in Pyeongchang, Republic of Korea, and the Tokyo 2020 Olympics [6].

Alcohol, soft drinks, energy drinks, and fast food companies have embraced this trend and are sponsoring esports-related events, encouraging poor dietary habits in esports players and spectators [7,8].

Inadequate dietary habits are linked to lower performance in a variety of cognitive areas, which might be explained in part by ethanol neurotoxicity, increased brain inflammation caused by high fat and high sugar consumption, and increased adiposity [9]. Contrarily, a healthier diet pattern (i.e., Mediterranean diet), composed mostly of whole grains, legumes, vegetables, fruit, and a modest amount of fish, dairy, and olive oil, is associated with optimal cognitive functioning in healthy adults [10,11,12].

Playing video games is associated with sedentarism, which increases weight gain and health risks [13,14]. Thus, esports players may require medical attention [15,16] and nutritional counseling [17] to optimize their health and performance. Nonetheless, the evidence for an association between esports practice and increased BMI is either null or mixed, and further research is required [18].

At least six investigations have assessed some aspect of the dietary habits of esports players [17,19,20,21,22,23].

In 2020, Rudolf and colleagues [17] applied an online survey to 1066 German video gamers and esports players and registered an average consumption of 2.7 (SD = 1.8) servings of fruit and vegetables, with only 11% of the sample meeting the WHO guidelines (≥400 g/day) [24].

Through an online survey, Huth (2021) [19] compared the dietary habits of German esports competitors (*n* = 241) with non-players (*n* = 98). The esports players expressed less concern with following a balanced diet (mean difference (MD) = −0.63; *p* < 0.001), ingested fewer portions of fruits and vegetables (MD = −1.15; *p* < 0.001), and drank fewer caffeinated beverages (MD = −1.32; *p* < 0.001) than non-players.

In 2023, Soffner and colleagues [20] characterized the dietary habits of 607 German video gamers and 210 esports players through an online food frequency questionnaire. The total sample averaged a daily consumption of 0.9 (SD = 1.0) of fruits, 1.7 (SD = 1.6) of vegetables, 0.5 (SD = 0.6) of red meat, 0.3 (SD = 0.4) of fast food, 0.2 (SD = 0.4) of chips and savory snacks, 0.2 (SD = 0.3) of sweet bakery products, 0.6 (SD = 0.8) servings of sweets, 0.9 (SD = 1.8) servings of soft drinks (180 mL), 0.4 (SD = 1.2) servings of light soft drinks (80 mL), 0.9 (SD = 1.4) servings of coffee (135 mL), and 0.2 (SD = 0.5) servings of energy drinks (50 mL). Esports players consumed a significantly greater daily amount of energy drinks (M = 0.2; SD = 0.6 servings) than video gamers (M = 0.1; SD = 0.4 servings; *p* = 0.02). Esports competitors also reported a statistically significant greater average consumption of fast food (9.1, SD = 11.6 vs. 7.0, SD = 10.0 servings/month; *p* < 0.01), and red meat (16.4, SD = 18.5 vs. 13.4, SD = 15.6 servings/month; *p* = 0.02) than video game players. Fast food (rho = 0.13; *p* < 0.01), energy drinks (rho = 0.14; *p* < 0.01), and soft drinks (rho = 0.14; *p* < 0.01) were positively (poorly) correlated with video game playing time.

In 2022, Szot and collaborators [21] assessed the dietary patterns of 233 Polish esports players through Food Frequency Questionnaires. Only 13.7% consumed fruit, and 11.2% ingested vegetables at least two or three times a day. Concerning unhealthy dietary patterns, the average daily consumption frequency for soft drinks was 3.4 (1.6), and 2.7 (1.4) for energy drinks. Three-quarters (75.5%) of the sample regularly consumed fried foods, roughly half (54.9%) ingested sweets, 28.3% had fast food, 28.3% ate red meat, and 28.7% consumed salty snacks.

In 2021, through a web-based survey, Ip et al. [22] assessed the dietary supplement consumption of 526 competitive video gamers, mostly United States citizens (77.0%). The most consumed DS was caffeine (58.1%), followed by vitamin B12 (3.8%), Yerba mate (2.3%), melatonin (1.5%), and Guarana (1.3%).

Goulart et al. (2023) [23] also assessed the dietary habits of USA esports players (*n* = 119) through food records and verified that the majority did not consume the recommended daily amount of vegetables (three cups), fruits (two cups), whole grains (one cup), dairy (three cups), and fiber (14 g/1000 kcal) described in the USDA Dietary Guidelines for Americans 2020–2025. Moreover, 83.5% surpassed the advised upper limits for sodium (2300 mg), 63.6% for cholesterol (300 mg), and 66.1% for saturated fat (10% of total energy intake).

Although esports has begun to draw the attention of the scientific community, health research [15] and nutrition for esports is still lacking, and, to date, no published study has yet assessed the dietary habits of Portuguese or Brazilian esports players. The inclusion of players of the Brazilian nationality was justified by the significant number of Brazilians living in Portugal, estimated to be 276,200 in 2020 [25], where several Brazilian esports players competed in Portuguese esports teams. Moreover, Brazil shares many traits, such as language, historical, commercial, and cultural ties with Portugal [26]. In addition, there were not any publications regarding the dietary habits of Brazilian esports players despite Brazil being one of the countries with the largest number of players, only surpassed by the USA and China [27], and despite being considered one of the countries in which esports has the greatest potential for expansion, especially in esports titles played on mobile devices [28].

This study aimed to characterize the esports gaming behaviors, degree of internet gaming disorder, and dietary habits of Portuguese and Brazilian esports competitors. Additionally, we intended to investigate associations between sociodemographic and esports-related variables and the level of adherence to the Mediterranean diet.

The finding presented in this original article may contribute to fill a knowledge gap in scientific literature and informing health professionals and individuals involved in esports on the dietary habits and gaming behaviors of Portuguese and Brazilian esports players. It may also serve as a foundation for public health institutes and esports organizations to develop strategic interventions to enhance the eating habits of these competitors.

## 2. Materials and Methods

A convenience sample of esports competitors was invited to respond to an anonymous, open online survey. The inquiry was conducted between 1 December 2022 and 30 June 2023. It was disseminated via email to esports organizations and through social media platforms, including Facebook, Twitter, Instagram, Discord, WhatsApp, and Twitch.

### 2.1. Inclusion/Exclusion Criteria

The study required participants to be between 18 and 69 years, have Portuguese or Brazilian nationality, and have played esports for at least six months. Individuals who did not fit these requirements were excluded. No rewards were offered for taking part in this investigation.

### 2.2. Survey Administration

A landing page was created on Google Sites, which contained two hyperlinks for two versions of the survey, generated on Google Forms: one specific for Portuguese and another for Brazilian participants. Google Analytics 4 was applied on the landing page to determine the number of unique site visitors, view rate, participation rate, and completion rate.

Each Google Form contained an easy-to-read summary of the study, a permission request, an email address for additional information requests, the anticipated conclusion time, and an invitation to participate. The Google Form, available as Supplementary Material (SM1), totaled 47 questions spread across seven pages. The survey was designed, applied, and reported accordingly to the CHERRIES checklist statement and was successfully pre-tested for functionality by the research team [29].

### 2.3. Questionnaires Included

The inquiry included:

Generic questions regarding nationality, sex, birth date, self-reported weight and height, education attainment, and professional occupation. Self-reported weight and height were used to calculate BMI (kg/m^2^), which was categorized based on the World Health Organization’s standards [30]. To minimize misreport bias, an equation was applied to predict BMI values based on self-reported weight and height [31].

Inquiries on gaming habits, namely, the number of hours of esports play per day, the number of competitions in the past 12 months, how many years and months playing esports, member of an esports team (yes or no), registration within an esports federation, level of professionalization (amateur, semi-professional, or professional), eGame that the respondent plays competitively, and their gaming rank.

The PREvención con DIeta MEDiterránea” (PREDIMED): It consists of fourteen multiple-choice questions that evaluate the typical ingestion of olive oil, spreadable fats, nuts, fruits, vegetables, legumes, fish or shellfish, meat, sugary drinks, wine, sweets, and stir-fried foods. The final score ranges from 0 to 14. We used three categories of Mediterranean diet adherence: low for values under 5, moderate for values between 6 and 9, and good for values above 10 [32,33]. Permission for PREDIMED (www.predimed.es, accessed on 2 October 2022) [32,33] utilization, including its Portuguese [34] and Brazilian version [35], was requested and obtained from the original authors. Additionally, from the data obtained in the PREDIMED questionnaire, we sought to determine the prevalence of individuals who did not comply with the World Health Organization’s fruit and vegetable consumption recommendations (at least 400 g/day) [24].

Fast-food and caffeine intake: An eight-item Likert scale was applied to assess breakfast omission, ranging from “Never” to “7 days”, and fast food and caffeinated beverages with a scale ranging from “Never“ to “4 or more per day”. The Brazilian caffeine content table (BraCaffT) was used to calculate the amount of caffeine that the respondents consumed from beverages [36].

A questionnaire consisting of five questions related to the consumption of DS, including types of DS consumed, reasons for DS consumption, sources of information, and place of acquisition.

The Internet Gaming Disorder Scale—Short-Form (IGDS9) characterizes the level of engrossment concerning online video gaming [37]. It has nine questions, with responses given on a 5-point Likert scale, with 1 denoting “Never”, and 5 denoting “Very often”. The overall score will vary between 9 and 45, with larger values indicating a higher risk of gaming addiction. The classification was based on the cut-off scores set by Severo et al. (2020): >16 points for moderate, and >21 points for high internet gaming disorder (IGD) risk [38]. Permission to use the IGDS9 was granted through email [37].

The anonymous electronic data gathered on Google Forms was downloaded and saved in a password-protected Excel file format (.xlsx) while complying with the EU’s General Data Protection Regulation [39].

### 2.4. Statistics

Microsoft Excel was used to organize, evaluate, and generate descriptive statistical data for the downloaded records. All statistical analysis was performed using IBM^®^ SPSS^®^ Statistics 29.0 for Windows (IBM Corp., Armonk, NY, USA). The significance value for all statistical tests was established at <0.05.

The following variables were generated and added to SPSS: “PREDIMED total score” (0 to 14); “portions of fruit and vegetables/day”; “<5 portions of fruits and vegetables/day” (no, yes); “portions of red and processed meat/day”; “<1 portion of red and processed meat/day” (no, yes); “No. of soft drinks/day”; “<1 soft drink/day” (no, yes); “No. of commercial pastries or sweets/week”; “<1 commercial pastry or sweet/week” (no, yes); “breakfast consumption frequency”; “frequency of fast food intake/week”; “IGDS9 total score” (0 to 45); No. of answers “Very often”; “nationality” (Portuguese, Brazilian); “sex” (male, female); “age” (18 to 69); “age—quartiles” (18 to 20, 21 to 23, 24 to 29, ≥30 y); “years of education” (0 to 16); “university education” (no, yes); “member of an esports team” (no, yes); “level of professionalization” (amateur, semi-professional or professional); “BMI” (kg/m^2^); “BMI—terciles” (<25, 25 to 30, >30 kg/m^2^); “federate athlete” (no, yes), “No. of esports titles played” (1 to 10); “No. of years playing esports”; “No. of years playing esports—terciles” (<5, 5 to 10, >10); “No. of hours of esports play/day”; “No. of hours of esports play/day—terciles” (≤2.5, 2.5 to 5, ≥5 h/day); “No. of competitions in the past 12 months”; “No. of competitions in the past 12 months—terciles” (0, 1–3, ≥4); “caffeine intake” (mg); “caffeine intake—terciles” (≤48, 48 to 224, >224 mg/day); “DS intake” (no, yes); “No. of DS ingested” (0 to 17); “DS consumed” (originally 40 answers, grouped into 8 categories: Proteins, Amino acids, Carbohydrates, Vitamin-mineral complexes, Isolated vitamins, Isolated minerals, Herbs, and Caffeine); “Reasons for using DS” (17 options); “Information sources about DS” (15 options); and “Place of acquisition of DS” (8 options).

Descriptive statistics were generated for each variable, and their normalities were assessed through skewness and kurtosis. Skewness and kurtosis exhibited a normal distribution (between −1 and 1) only for the “PREDIMED total score”, “IGDS9 total score”, “portions of red and processed meat/day”, “No. of soft drinks/day”, and “No. of commercial pastries or sweets/week” variables.

Data from Portuguese and Brazilian players were compared using Student’s *t*-test for “PREDIMED total score”, “IGDS9 total score”, “portions of fruit and vegetables/day”, “portions of red and processed meat/day”, “No. of soft drinks/day”, “No. of commercial pastries or sweets/week”, and Mann–Whitney’s U test for “level of professionalization”, “age”, “BMI”, “years of education”, “No. of hours of esports play/day”, “No. of competitions in the past 12 months”, “No. of years playing esports”, “No. of esports titles played”, “breakfast consumption frequency”, “frequency of fast food intake/week”, “caffeine intake”, and “No. of DS ingested”. Crosstabulations and Fisher’s exact test were used to describe the relationship between the “sex”, “<5 portions of fruits and vegetables/day”, “<1 portion of red and processed meat/day”, “<1 soft drink/day”, “<1 commercial pastry or sweet/week”, “member of an esports team”, “DS intake”, and “federate athlete” variables and “nationality”.

## 3. Results

The Google website received 2039 unique visitors overall; 1566 (76.8%) of them navigated to the Google form that included the survey, and 711 (45.4%) of those completed it.

A total of 132 responses were rejected: four for stating a nationality other than Portuguese or Brazilian, nine for not having played esports for at least six months, 105 for being less than 18 years old, one for reporting being older than 69, two for not reporting the esports title they played competitively, one for reporting zero hours of esports play per day, and ten for providing nonsensical answers. As a result, the information from 579 individuals was included and examined in this study.

### 3.1. Socio-Demographic Characteristics

A little more than half were Brazilian (52.7%; *n* = 305), and the remaining were of Portuguese nationality. This sample was predominantly male (91.4%; *n* = 529), aged between 18 and 29 years (75.0%; *n* = 434). The median number of years of schooling was 14 years, with about one-third of the sample (37.0%; *n* = 214) reporting having completed university education. Concerning employment status, 43.9% (*n* = 254) of the sample reported being an employee, whereas about one fifth (21.1%; *n* = 122) reported being a student worker, and another fifth (22.3%; *n* = 129) a student (Table 1).

About half of the sample was classified as having a normal weight (48.9%; *n* = 283, whereas 46.6% (*n* = 270) were overweight.

Compared to Portuguese, Brazilian players had a significantly lower “age” (*p* < 0.001), fewer “years of education” (*p* = 0.010), and a higher “BMI” (*p* = 0.005). The proportion of males and females was not different among Brazilian and Portuguese players (*p* = 0.104).

### 3.2. Esports-Related Variables

The respondents reported playing esports for 8.1 (SD = 5.1) years, practicing esports 4.4 (SD = 3.2) hours a day, and participating in a total of 7.6 (SD = 10.4) competitions in the past 12 months on average. Most respondents reported being amateur esports players (62.2%; *n* = 360), whereas the remaining self-classified as semi-professional or professional players. About a quarter (25.9%, *n* = 150) stated they were a member of an esports league or federation (Table 2).

The Brazilians played esports for a significantly higher number of hours per day (*p* < 0.001), had been playing esports for more years (*p* = 0.011), played a higher number of esports titles (*p* < 0.001), and reported a higher level of professionalization (*p* = 0.011) than the Portuguese players. Additionally, a significantly lower proportion of Brazilians reported being members of an esports team (*p* < 0.001) or federation (*p* < 0.001).

Most participants played First Person Shooter games (70.5%; *n* = 408), with the Multiplayer Online Battle Arena genre being the second most popular (35.1%; *n* = 203), followed by racing simulators (26.1%; *n* = 151), and other sports simulators (12.4%; *n* = 72) games.

The specific esports titles with the most players were League of Legends (25.9%; n = 150), Counter-Strike: Global Offensive (24.7%; *n* = 143), Valorant (17.4%; *n* = 101), and FIFA 2022/23 (12.3%; *n* = 71).

The respondents averaged a total score of 18.9 (SD = 6.8) on the IGDS9 scale, with 2.6% (*n* = 15) accumulating a total score of 36 points or higher. About a quarter (26.8%; *n* = 155) of the total sample answered “Very often” on at least one of the eight questions included in the IGDS9 (Table 3).

Brazilian esports players displayed a significantly higher overall IGDS9 score (*p* < 0.001) than Portuguese players.

### 3.3. Dietary Habits

More than half of our sample (53.7%; *n* = 311) classified as having poor adherence to the Mediterranean diet pattern (PREDIMED), and most respondents (84.5%; *n* = 489) stated eating less than five servings of fruits and vegetables per day. Over half (63.4%; *n* = 367) stated consuming fast food, 70.1% (*n* = 406) sweets or commercial pastries, 65.8% (*n* = 381) coffee, and almost a quarter (23.0%; *n* = 133) reported drinking energy drinks on one or more occasions per week. The consumption of caffeine derived from caffeinated drinks averaged 179 (SD = 208) mg per day (Table 4).

Additionally, more than three-thirds of the respondents (78.1%; *n* = 452) ate one or more servings of red/processed meat, and 40.9% (*n* = 237) consumed one or more soft drinks per day.

When comparing the data of Portuguese players with those of Brazilian players, Portuguese players displayed a statistically significant higher PREDIMED total score (*p* < 0.001), a higher consumption of fruit and vegetables (*p* < 0.001), and a lower consumption of soft drinks (*p* < 0.001). No significant differences were observed for red and processed meat (*p* = 0.814) and commercial pastries and sweets (*p* = 0.424).

Significant relationships were observed between nationality and the number of soft drinks (*p* < 0.001) and portions of red and processed meat consumed per day (*p* < 0.016), corresponding to a greater consumption of red and processed meat in the Portuguese and a greater intake of soft drinks in the Brazilians. No significant associations were registered between nationality and the number of portions of fruits and vegetables ingested daily (*p* = 0.168) and the weekly consumption of commercial pastries or sweets (*p* = 0.413).

Regarding the breakfast meal, almost a quarter of our sample never ate breakfast (23.0%; *n* = 133) and almost half (47.5%; *n* = 275) skipped breakfast on three or more days per week (Table 5).

No statistically significant differences were observed regarding breakfast consumption frequency (*p* = 0.809) and caffeine intake (*p* = 0.652) between the Brazilian and Portuguese players; however, Brazilian players did consume fast food more frequently than Portuguese players (*p* = 0.002).

### 3.4. Dietary Supplements Intake

**Figure 1:**
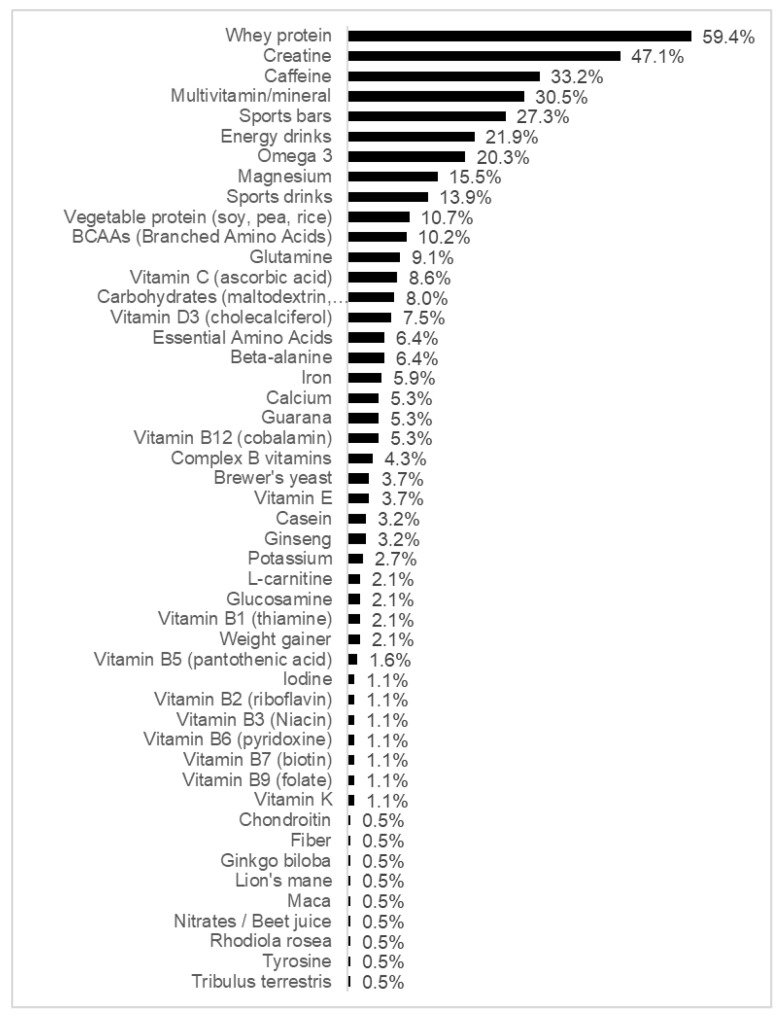
Types of dietary supplements consumed.

Almost a third of the total sample (32.3%; *n* = 187) stated consuming DS in the past 12 months (Table 6). The most consumed DS were whey protein (59.4%; *n* = 111), creatine (47.1%; *n* = 88), caffeine (33.2%; *n* = 62), and multivitamin-mineral (30.5%; *n* = 57) (Figure 1).

No statistically significant differences were found between Brazilian and Portuguese players regarding DS consumption in the past 12 months (*p* = 0.213) or the number of DS ingested (*p* = 0.194).

The top five reasons for using DS were: to improve athletic performance (84.5%; *n* = 158), prevent health problems or treat illness/injury (58.8%; *n* = 110), improve cognitive performance (56.7%; *n* = 106), gain muscle mass (51.9%; *n* = 97), and increase energy/decrease fatigue (49.7%; *n* = 93) (Table 7).

Nutritionists (42.2%; *n* = 79), medical professionals (23.5%; *n* = 44), athlete recommendations (19.8%; *n* = 37), and personal trainers (19.3%; *n* = 36) were the most frequently cited sources of DS related knowledge (Table 8).

Regarding adherence to the Mediterranean dietary pattern, represented by PREDIMED total score, when encompassing data relating to both nationalities, the univariate ANOVA returned statistical significance for nationality, sex, DS intake, and IGDS9 total score (Table 9). Portuguese nationality was associated with a higher PREDIMED total score (*p* < 0.001, eta_p_^2^ = 0.052), as well as being a female (*p* = 0.034, eta_p_^2^ = 0.008). Not having consumed DS in the past 12 months (*p* < 0.001, eta_p_^2^ = 0.029), and a higher IGDS9 total score (*p* = 0.002, eta_p_^2^ = 0.017) were associated with a lower PREDIMED total score.

When analyzing the data for Portuguese and Brazilians separately, the univariate ANOVA returned distinct results. The relationship with sex was not significant in either country, whereas DS intake remained significant in both groups, with the Brazilian (*p* = 0.004, eta_p_^2^ = 0.029) and Portuguese (*p* = 0.010, eta_p_^2^ = 0.026) non-users of DS presenting a lower PREDIMED total score. The IGDS9 total score remained significant only for the Brazilian, associating with a lower PREDIMED total score (*p* = 0.018, eta_p_^2^ = 0.020). In addition, a significant relationship regarding the level of professionalization emerged for the Portuguese players (*p* = 0.010, eta_p_^2^ = 0.036), with amateurs (estimated marginal mean (EMM) = 6.664) presenting a higher PREDIMED total score compared to semi-professionals (EMM = 5.886) and professionals (EMM = 5.263).

## 4. Discussion

### 4.1. BMI

A higher BMI or adiposity has been associated with an increased risk for a variety of health problems and greater morbidity [40]. Increased neuroinflammation, gray matter atrophy in specific brain areas, diminished cognitive capacity [9], and lower esports performance [41], were also reported.

According to data provided by the Brazilian Institute of Geography and Statistics (IBGE), by 2019, 60.3% of Brazilian adults (≥18 years) were overweight, and 25.9% were obese [42]. In Portugal, more than half (58.1%) of the adults exhibited excessive adiposity. 36.5% were classified as being overweight and 21.6% as obese, according to a national inquiry conducted between 2015 and 2026 (IAN-AF 2015-16) [43].

In comparison, 46.6% of the participants in our study had excess weight. More specifically, 28.0% were categorized as pre-obese and 18.7% as obese. This could have been due to our younger sample compared with that of studies conducted in reference populations [42,43], as increased age is associated with weight gain in adults [44].

When stratifying by age groups, in comparison with a reference population, our sample of Brazilian esports players presented lower pre-obesity prevalence from 18 to 24 years old (20.4% vs. 33.3%) and from 25 to 39 years (36.0% vs. 57.6%). However, they presented a higher prevalence of obesity in the same age groups: 18–24 years (18.3% vs. 10.7%) and 25–39 years (33.3% vs. 23.7%) [42].

Our sample of Portuguese esports players presented a higher prevalence of pre-obesity in those aged between 18–24 years (24.4% vs. 19.1%) and those aged 35–44 years (48.7% vs. 37.1%), but not among the subgroup of 25–34 years (27.0% vs. 31.8%) compared to a reference population. The prevalence of obesity was also slightly higher for players aged between 18–24 years (6.5% vs. 6.3%) and 25–34 years (14.0% vs. 10.8%), but not among those aged 35–44 (15.4% vs. 20.2%) [43].

These findings imply that esports participation may be linked to a higher prevalence of pre-obesity and obesity in particular age groups among Brazilian and Portuguese esports participants.

Furthermore, we observed significantly higher BMI values in Brazilian compared to Portuguese esports players (*p* = 0.005), which may have been related to their significantly lower PREDIMED total score (*p* < 0.001), lower consumption of fruit and vegetables (*p* < 0.001), higher consumption of soft drinks (*p* < 0.001), and higher consumption frequency of fast food (*p* < 0.002).

### 4.2. Internet Gaming Disorder

Internet gaming disorder is a disorder that implies an uncontrollable obsessive dedication to online video games, resulting in adverse physical and psychological consequences. It is associated with interpersonal difficulties, sleep disturbances, low dietary quality, and exaggerated caffeine intake [45].

In a study conducted in Brazil in 2017, Severo et al. (2020) applied the IGDS9 questionnaire to 555 students (57.5% male) with a mean age of 20.3 (SD = 5.4) years who had played video games in the previous 12 months. They found that 38.2% of the volunteers had a moderate (score 16 to 20) and 13.5% had a high risk (score > 21) of gaming disorder [46]. When applying the same cut-off points, our research uncovered a higher percentage of individuals with a score higher than 16 (56.8%) and 21 (30.6%). This may have been due to our study including a sample with a higher percentage of males (91.4%) and dedicating longer periods to video games, which have been reported as a risk factor for IGD by Severo et al. (2020) [46]. We may also have a high proportion of multiplayer online battle arena (MOBA) game players, which seem to be at a higher risk for IGD, compared to first-person shooter (FPS) players [47].

In comparison, Ip et al. (2021) surveyed 526 adult video gamers in the USA (23.9; SD = 4.9 years) and reported that 2.3% of their total sample formally met the diagnostic criteria for IGD [22]. Of note, these investigators used a score ≥ 36 as an indicator of IGD, and, using that same value, we registered a prevalence of 2.6% (*n* = 15), which represented a slightly higher prevalence.

Regarding the two nationalities covered in our study, we found significantly higher IDGS9 total score values in Brazilian esports players compared to Portuguese ones (*p* < 0.001).

### 4.3. Mediterranian Diet Adherence

The IAN-AF 2015-16, conducted in Portugal, also applied the Mediterranean Diet Score and revealed that 15.7% of the Portuguese adult population has a high adherence, 50.0% moderate and 34.3% low adherence to the Mediterranean dietary pattern [43]. In comparison, we report that a mere 3.6% of our total sample was classified as having good or very good adherence, 42.7% moderate to fair adherence, and 53.7% with weak adherence in the PREDIMED score.

Our Brazilian sample had a significantly lower PREDIMED total score compared to Portuguese respondents (*p* < 0.001), which may have been related to differences in public food policy, food culture, and food availability between the two countries [48].

Our analysis revealed that DS consumption was associated with a higher PREDIMED score, whereas a higher IGD total score and a higher level of professionalization correlated with a lower PREDIMED score. As seen in other studies, those that consumed DS were probably more health-conscious people who cared more about following a healthy diet [49].

Other research also demonstrated an association between a higher degree of IGD and a higher prevalence of inadequate dietary habits [45]. The attempt to prolong game periods can lead to the choice of more convenient but less nutritionally adequate food options, such as fast food, and longer periods of distraction can decrease the awareness of what is being ingested and decrease the ability to regulate food ingestion [50]. It is also likely that increasing professionalization, implying a higher commitment to esports, results in less concern with following a healthy eating pattern.

### 4.4. Fruits and Vegetables

Fruits and vegetables provide a host of benefits that extend beyond their vitamin, mineral, and fiber contents. These foods include a range of bioactive compounds that benefit health and decrease all-cause mortality [51] and have been associated with optimal cognitive performance in young adults [52] and delayed cognitive decline in older individuals [53].

According to the IBGE, in 2013, 62.7% of Brazilian adults aged 18 years or over did not ingest the recommended amount of fruits and vegetables [42]. As reported by the IAN-AF 2015-16 research, the average daily consumption of “Fruit, vegetables, and legumes” by the Portuguese population is 312 g/day, and 57% of Portuguese adults (18 to 64) do not consume the minimum amount of vegetables and fresh fruits recommended by the WHO (400 g/day) [24,43].

Our study uncovered even higher values of non-compliance, with 84.5% of the total sample not reaching the recommended five portions of fruits and vegetables per day. Moreover, our sample of Portuguese players reported significantly higher average consumption of fruit and vegetables than the Brazilian players (*p* < 0.001).

### 4.5. Breakfast Omission

According to a meta-analysis, skipping breakfast ≥3 days per week increases the risk for overweight/obesity by 11% (95% CI: 4 to 19%) compared to ≤2 days per week [54]. It also correlates with higher visceral adiposity [55], decreased sleep quality [56], lower intake of vitamins and minerals [57], fatigue exacerbation, diminished attention, inferior academic performance [58], and inhibitory control [59].

As the brain depends mainly on glucose as a fuel source, with glucose requirements increasing by up to 12% in the context of cognitively demanding activities [9], having a breakfast that includes carbohydrates-containing foods may be crucial for the optimize cognitive performance of esports players that regularly includes intensive esports training sessions and competitions on their schedules.

In a 2007 study conducted in Brazil, 30% of 100 students (19 to 39 years) at a university in the city of Campinas reported skipping breakfast [60]. In another study, conducted in 2002, which involved 185 students (18 and 19 years old) of a Brazilian public university, 37% stated omitting breakfast [61]. According to the IAN-AF 2015-16 national survey, 6.4% of Portuguese adults regularly omit breakfast, with a higher prevalence of breakfast skipping among adolescents (8.4%) [43].

Regarding this topic, 23% of our respondents admitted never having breakfast, whereas 47.5% reported skipping breakfast on three or more occasions per week. No statistically significant differences were observed regarding the median frequency of breakfast intake when comparing Brazilian to Portuguese esports players (*p* = 0.809).

### 4.6. Fast Food Intake

In addition to being associated with increased weight and more prevalence of health problems [62], higher fast food consumption correlated with lower executive function and visual memory capacity in university students [63].

According to data collected by the IBGE, in 2019, 6.6% of Brazilians aged 18 or over regularly replaced at least one meal with sandwiches, snacks, or pizza [42]. According to the IAN-AF 2015-16, Portuguese adults consumed fast food (snacks, fried foods, and pizza) on 12.5% of the days and registered an average consumption of 23.3 g of fast food per day [43].

Our study revealed that 39.6% of respondents ingested fast food on one, 20.4% on two to four, and 3.5% on five or more occasions per week. Despite the distinct methodologies applied in these studies, we can infer that fast food consumption was more prevalent in our sample of esports players compared to a reference population. Additionally, we registered a significantly higher consumption of fast food among players of Brazilian nationality compared to Portuguese nationality (*p* = 0.002).

### 4.7. Red Meat

Red and processed meat is linked to an increased risk of colon cancer, with a 17% (95% CI: 5 to 31%) higher relative risk for daily consumption of 100 g of red meat and 18% for 50 g of processed meat [64].

According to the 2015-16 IAN-AF survey, about a quarter (25.5%) of Portuguese adults eat between 100 and 150 g of red meat daily [43]. In divergence, our study found much higher results (78.1%) for daily consumption of an amount equal to or greater than 100 to 150 g of red or processed meat.

Although we did not observe significant differences between the two nationalities regarding the number of servings of red and processed meat eaten per day (*p* = 0.814), we found that a higher proportion of Brazilian players ate less than one serving of red and processed meat per day, compared to the Portuguese (*p* = 0.016).

### 4.8. Soft Drinks

Soft or sugary drink consumption has been associated with increased body weight [65], poorer oral health [66], lower consumption of milk and calcium [67], inferior sleep quality [68], and decreased cognitive performance, specifically in the working memory, visual scanning and tracking, and executive function domains [69].

As stated by the IBGE in 2019, 9.2% of Brazilians ingest soft drinks regularly [42]. A national survey (VIGITEL Brasil 2018) found that the frequency of adults who drink soft drinks on five or more days of the week ranges from 6.0% in João Pessoa to 23.0% in Porto Alegre [70]. According to the IAN-AF 2015-16, Portuguese adults (18 to 64 years) consume soft drinks on 15.8% of the days, at an average of 99.6 mL per day.

In comparison, we found that 40.9% of our total sample consumed one or more soft drinks daily, with a significantly higher average daily consumption of soft drinks in the Brazilian compared to the Portuguese players (*p* < 0.001).

### 4.9. Caffeine Intake

Regular light to moderate caffeine intake has been associated with a lower risk of all-cause mortality, cardiovascular mortality, and a decreased risk of developing conditions such as obesity, type 2 diabetes mellitus, depression, dementia, and some types of cancer [71,72]. However, caffeine also increases the risk of harmful events, including decreased sleep duration and quality, increased anxiety, and nervousness [73].

According to a national survey conducted between 2008 and 2009, which included individuals aged 10 years and older, Brazilians ingest an average of 115.7 mg of caffeine per day from foods and beverages. For individuals aged between 19 and 30 years, the mean intake was 110.2 mg/day, and 125.2 mg/day for those aged 31 to 50 years [74]. Based on data collected in a national survey carried out in 2009 in Portugal, Pinhão and colleagues found that, on average, Portuguese people aged 18 to 29 ingested 148 mg, and those with 30 to 44 years consumed 220 mg of caffeine per weekday from food and beverages [75].

We registered a median daily consumption of 112 mg of caffeine, with 65.8% ingesting coffee and 23.0% consuming energy drinks at least once a week. In comparison, Ip et al. (2021), who surveyed 526 adult video gamers in the USA, reported that 79.5% and 45.1% regularly consumed caffeinated and energy drinks, respectively [22]. Additionally, Skarupova and colleagues (2018) observed that 74.2% of 3952 Czech and Slovak players of massively multiplayer online games aged 11 to 59 consumed caffeine while playing [76].

Although we did not observe significant differences between the two nationalities regarding caffeine consumption (*p* = 0.652), we did observe that Brazilian players’ consumption of coffee (*p* = 0.036) and energy drinks (*p* < 0.001) was significantly higher than that of Portuguese players.

### 4.10. Dietary Supplements Intake

Two studies characterized DS consumption in Brazilian urban areas. A study conducted in São Paulo between 2007 and 2008, which involved 865 individuals aged between 12 and over 60, found that only 6.35% consumed DS, mainly vitamins and minerals (41%), protein (5%), branched amino acids (5%), nutrient-enriched foods (4%), energy drinks (4%), meal replacements for weight loss (2%), and others (5%) [77]. Another investigation took place in Brasília between 2016 and 2017 and involved 506 volunteers aged 20 years or over, of which 35% used DS. The most consumed were vitamins and minerals (55%), proteins (44%), omega-3 (21%), creatine (6%), caffeine (5%), and energizers (2%) [78].

The use of DS in the previous 12 months has been reported by 29.2% of the Portuguese adult population (18 to 64 years). The most consumed DS were multivitamin complexes (33.1%), mineral salts (33.1%), and isolated vitamins (21.3%), such as folic acid and vitamin C, and other categories of DS (15.5%), including whey protein and omega 3 fatty acids [43].

Our study unveiled a higher prevalence of DS consumption (32.3%) than those reported by the studies conducted in the Brazilian and Portuguese populations. The most consumed DS were whey protein (59.4%), creatine (47.1%), caffeine (33.2%), and multivitamin-mineral (30.5%). The differences in DS consumption may have been related to our sample being mostly of young adult age, in which the majority (84.5%) sought to improve some aspect of athletic performance or augment their muscle mass (51.9%).

We did not observe statistically significant differences regarding the proportion of consumers of DS (*p* = 0.213) and the number of DS ingested (*p* = 0.194) between our sample of Brazilian and Portuguese players.

Our study also uncovered a significantly higher prevalence of caffeine supplements consumption compared to a reference population, and even higher than those reported by Ip et al. (2021), who surveyed 526 adult video gamers in the USA and found that 12.1% (*n* = 27) had consumed caffeine pills in the previous 12 months [22]. This higher caffeine consumption through drinks and DS could be related to the ultra-competitive nature of esports, the relatively high number of hours dedicated to video gaming, or sleep perturbations. As about half of our sample admitted using DS to boost their cognitive performance (56.7%) or increase energy/decrease fatigue (49.7%); they may have resorted to caffeine for that purpose.

Although their efficacy has not yet been assessed in esports players, we find it intriguing that two of the DS with the highest nootropic potential in young adults, tyrosine [79] and Bacopa monnieri [80], were seldom used by our respondents.

### 4.11. Limitations

This survey was conducted during the winter and spring, between December and June, and, therefore, the nutritional data obtained only concerned this period of seven months of the year [81].

Although considered valid measurement tools, the retrospective nature of the questionnaires applied in this investigation implies the possibility of memory errors [82]. Furthermore, web-based surveys may be vulnerable to substantial bias due to the participant’s self-selection (volunteer effect) [29]. Due to the anonymous nature of the survey, the possibility of duplicate responses remains.

When available, we compared our data with those reported by other studies that used different methodologies, which limited comparisons.

We did not characterize the city, district, or state of residence, which may modulate the eating behaviors and lifestyles of the population [70]. Justified by the need to keep the questionnaire reasonably short and quick to complete, we restricted our caffeine intake assessment to the most commonly consumed sources of caffeine (coffee, tea, cola, and energy drinks) [73] and did not assess physical activity level or sleep-related variables.

The Brazilian caffeine content table (BraCaffT) [36], developed in Brazil and specific for the Brazilian population, was used to determine the amount of caffeine ingested through beverages, which may translate into a less accurate assessment of caffeine consumption for Portuguese esports players.

### 4.12. Final Remarks

The young adult age is a crucial period of human development in which inappropriate eating habits can have irreversible or difficult-to-reverse negative health consequences, which will extend throughout adult life [83]. This topic is especially relevant for esports players, as nutrition affects cognitive performance, on which esports players depend for prime performance during training and competition [9].

This study adds to the evidence that most Portuguese and Brazilian esports players have a low level of adherence to a healthy eating pattern. It is necessary to promote healthier dietary habits in this population while involving esports organizations and public health institutions.

Compared to the Portuguese, Brazilian players are more vulnerable and more in need of this type of intervention since they have a lower average age, fewer years of education, a higher level of professionalism, a higher number of hours dedicated to esports play, higher IGD score, higher unemployment, and a higher degree of dietary inadequacy, as demonstrated by lower PREDIMED scores, lower consumption of fruit and vegetables, and higher coffee, energy drinks, soft drinks, and fast-food consumption.

This research may serve as a basis for the development of strategies to promote appropriate eating habits among Portuguese and Brazilian esports players.

### 4.13. Future Directions

Future investigations should apply food diaries to Portuguese and Brazilian esports players to assess their eating habits more accurately, including fluid consumption, for further insight regarding possible dietary inadequacies, including micronutrient intake deficits.

Additionally, one could test the hypothesis that greater adherence to a healthy dietary pattern, such as the Mediterranean diet, is associated with higher cognitive and video gaming performance in esports players.

## 5. Conclusions

Our sample presented a low adhesion to the Mediterranean dietary pattern, low compliance to the WHO recommended amount of vegetables and fruit, and high consumption of fast food, red meat, soft drinks, and dietary supplements, including caffeine-based supplements.

In comparison with the Brazilians, Portuguese esports players are older, have a lower BMI, a lower IGD score, a higher adhesion to the Mediterranean diet, higher consumption of fruit and vegetables, lower consumption of soft drinks and fast-food, a higher educational level, a higher number of members of esports teams and federate players, play esports fewer hours a day, have been playing esports for fewer years, play a lower number of esports titles, and display a lower level of esports professionalization.

For both nationalities, the consumption of dietary supplements was associated with a higher adhesion to the Mediterranean diet and a higher degree of gaming disorder correlated with a lower adhesion to this dietary regimen. For the Portuguese players, a higher level of professionalization was associated with a lower adhesion to the Mediterranean diet.

We infer that Portuguese and Brazilian esports players adhere to an unbalanced diet. We advocate for the implementation of initiatives to promote healthy eating habits in this community, preferably with the support of esports organizations.

## Figures and Tables

**Table 1:** Socio-demographic characteristics.

	Total	Brazilian	Portuguese	
	*n* (%)	*n* (%)	*n* (%)	*p*
**Sex**				0.104 ^F^
Male	529 (91.4)	273 (89.5)	256 (93.4)	
Female	50 (8.6)	32 (10.5)	18 (6.6)	
**Age**, years (Mdn; P25, P75)	24 (20, 30)	23 (20, 28)	26 (22, 31)	<0.001 ^M^
18 to 29 y	434 (75.0)	250 (82.0)	184 (67.2)	
30–39 y	110 (19.0)	47 (15.4)	63 (23.0)	
40–49 y	28 (4.8)	6 (2.0)	22 (8.0)	
50–59 y	6 (1.0)	2 (0.7)	4 (1.5)	
60–69 y	1 (0.2)	0 (0.0)	1 (0.4)	
**BMI**, kg/m^2^ (Mdn; P25, P75)	24.6 (22.3, 28.5)	25.2 (22.5, 30.2)	24.2 (22.2, 26.7)	0.005 ^M^
Underweight (<18.5 kg/m^2^)	26 (4.5)	16 (5.2)	10 (3.6)	
Normal range ([18.5; 25.0] kg/m^2^)	283 (48.9)	133 (43.6)	150 (54.7)	
Pre-obesity ([25.0; 30.0] kg/m^2^)	162 (28.0)	79 (25.9)	83 (30.3)	
Obesity class I ([30.0; 35.0] kg/m^2^)	65 (11.2)	48 (15.7)	17 (6.2)	
Obesity class II ([35.0; 40.0] kg/m^2^)	43 (7.4)	29 (9.5)	14 (5.1)	
**Education**, years (Mdn; P25, P75)	14 (12, 16)	13 (12, 16)	14 (12, 16)	0.010 ^M^
0–9 y	32 (5.5)	14 (4.6)	18 (6.6)	
10–12 y	189 (32.6)	97 (31.8)	92 (33.6)	
13–14 y	124 (21.4)	83 (27.2)	41 (15.0)	
14–15 y	20 (3.5)	20 (6.6)	0 (0.0)	
≥16 y	214 (37.0)	91 (29.8)	123 (44.9)	
**Employment status**				<0.001 ^F^
Student	129 (22.3)	64 (21.0)	65 (23.7)	
Working Student	122 (21.1)	89 (29.2)	33 (12.0)	
Worker	254 (43.9)	105 (34.4)	149 (54.4)	
Unemployed	60 (10.4)	36 (11.8)	24 (8.8)	
Other ^a^	14 (2.4)	11 (3.6)	3 (1.1)	

Abbreviations: Mdn = median; P25 = 25th percentile; P75 = 75th percentile; BMI = Body mass index; ^M^ = Mann–Whitney U test; ^F^ = Fisher’s exact test. ^a^ Other includes: retired, permanently disabled, student, domestic worker, or performing mandatory military service.

**Table 2:** Data on esports-related variables.

	Total	Brazilian	Portuguese	
	*n* (%)	*n* (%)	*n* (%)	*p*
**Level of professionalization**				<0.001 ^M^
Professional	72 (12.4)	58 (19.0)	14 (5.1)	
Semi-professional	147 (25.4)	72 (23.6)	75 (27.4)	
Amateur	360 (62.2)	175 (57.4)	185 (67.5)	
Federated athlete	150 (25.9)	48 (15.7)	102 (37.2)	<0.001 ^F^
Member of an esports team	89 (15.4)	89 (29.2)	143 (52.2)	<0.001 ^F^
Reported eGame rank	423 (73.1)	245 (80.3)	178 (65.0)	<0.001 ^F^
Competed in the past 12 months	374 (64.6)	187 (61.3)	187 (68.2)	0.083 ^F^
No. of competitions in the past 12 months (Mdn; P25, P75)	2 (0, 5)	2 (0, 5)	2 (0, 5)	0.241 ^M^
No. years playing esports (Mdn; P25, P75)	8.0 (5.0, 12.0)	8.0 (5.0, 12.2)	7.0 (4.0, 11.0)	0.011 ^M^
No. hours of esports play/day (Mdn; P25, P75)	3.5 (2.0, 6.0)	4.0 (3.0, 6.8)	3.0 (2.0, 4.0)	<0.001 ^M^
No. esports titles played (Mdn; P25, P75)	3 (1, 5)	5 (5, 5)	1 (1, 2)	<0.001 ^M^

Abbreviations: Mdn = median; P25 = 25th percentile; P75 = 75th percentile; ^M^ = Mann–Whitney’s U test; ^F^ = Fisher’s exact test.

**Table 3:** Data on the IGDS9 results.

	Total	Brazilian	Portuguese	
	*n* (%)	*n* (%)	*n* (%)	*p*
**IGDS9 total score** (m; sd)	18.9 (6.8)	20.9 (7.1)	16.7 (5.7)	<0.001 ^T^
IGDS9 total score > 16	329 (56.8)	206 (67.5)	123 (44.9)	
IGDS9 total score > 21	177 (30.6)	129 (42.3)	48 (17.5)	
IGDS9 total score ≥ 36	15 (2.6)	12 (3.9)	3 (1.1)	

Abbreviations: m = mean; sd = standard deviation; ^T^ = independent sample’s *t*-test.

**Table 4:** Data on the PREDIMED related variables.

	Total	Brazilian	Portuguese	
	*n* (%)	*n* (%)	*n* (%)	*p*
**PREDIMED total score** (m; sd)	5.4 (2.2)	4.7 (2.1)	6.1 (2.1)	<0.001 ^T^
Weak adherence (≤5)	311 (53.7)	205 (67.2)	106 (38.7)	
Moderate to fair adherence (6 to 9)	247 (42.7)	96 (31.5)	151 (55.1)	
Good or very good adherence (≥10)	21 (3.6)	4 (1.3)	17 (6.2)	
**Portions of fruits and vegetables/day** (m; sd)	2.5 (1.9)	2.3 (1.9)	2.8 (1.9)	<0.001 ^T^
<5 portions/day	489 (84.5)	264 (86.6)	225 (82.1)	0.168 ^F^
≥5 portions/day	90 (15.5)	41 (13.4)	49 (17.9)	
**Portions of red and processed meat/day** (m; sd)	1.6 (1.2)	1.6 (1.2)	1.6 (1.1)	0.814 ^T^
<1 portion/day	127 (21.9)	79 (25.9)	48 (17.5)	0.016 ^F^
≥1 portion/day	452 (78.1)	226 (74.1)	226 (82.5)	
**No. of soft drinks/day** (m; sd)	0.8 (1.2)	1.0 (1.3)	0.7 (1.1)	<0.001 ^T^
<1 drink/day	342 (59.1)	159 (52.1)	183 (66.8)	<0.001 ^F^
≥1 drink/day	237 (40.9)	146 (47.9)	91 (33.2)	
**No. of commercial pastries or sweets/week** (m; sd)	2.2 (2.1)	2.2 (2.3)	2.1 (2.0)	0.424 ^T^
<1 occasion/week	173 (29.9)	96 (31.5)	77 (28.1)	0.413 ^F^
≥1 occasion/week	406 (70.1)	209 (68.5)	197 (71.9)	

Abbreviations: m = mean; sd = standard deviation; ^T^ = independent sample’s *t*-test; ^F^ = Fisher’s exact test.

**Table 5:** Data on breakfast, fast food, and caffeinated drinks.

	Total	Brazilian	Portuguese	
	*n* (%)	*n* (%)	*n* (%)	*p*
**Breakfast consumption frequency**				
Never	133 (23.0)	75 (24.6)	58 (21.2)	
One day/week	15 (2.6)	5 (1.6)	10 (3.6)	
Two days/week	46 (7.9)	22 (7.2)	24 (8.8)	
Three days/week	53 (9.2)	24 (7.9)	29 (10.6)	
Four days/week	28 (4.8)	16 (5.2)	12 (4.4)	
Five days/week	57 (9.8)	30 (9.8)	27 (9.9)	
Six days/week	34 (5.9)	17 (5.6)	17 (6.2)	
Seven days/week	213 (36.8)	116 (38.0)	97 (35.4)	
Breakfast consumption frequency, days per week (Mdn; P25, P75)	5 (1, 7)	5 (1, 7)	5 (0, 5)	0.809 ^M^
**Fast food intake**				
Never	212 (36.6)	101 (33.1)	111 (40.5)	
1 occasion/week	229 (39.6)	114 (37.4)	115 (42.0)	
2 to 4 occasions/week	118 (20.4)	74 (24.3)	44 (16.1)	
5 to 6 occasions/week	12 (2.1)	10 (3.3)	2 (0.7)	
1 occasion/day	6 (1.0)	4 (1.3)	2 (0.7)	
2 occasions/day	2 (0.3)	2 (0.7)	0 (0.0)	
Fast food intake, occasions per week (Mdn; P25, P75)	1 (0, 1)	1 (0, 3)	1 (0, 1)	0.002 ^M^
Caffeine from caffeinated drinks, mg/day (Mdn; P25, P75)	112 (27, 256)	112 (32, 241)	116 (22, 291)	0.652 ^M^
**Coffee intake**				0.036 ^M^
Never	198 (34.2)	117 (38.4)	81 (29.6)	
1 occasion/week	35 (6.0)	18 (5.9)	17 (6.2)	
2 to 4 occasions/week	58 (10.0)	23 (7.5)	35 (12.8)	
5 to 6 occasions/week	40 (6.9)	24 (7.9)	16 (5.8)	
1 occasion/day	61 (10.5)	38 (12.5)	23 (8.4)	
2 occasions/day	78 (13.5)	36 (11.8)	42 (15.3)	
3 occasions/day	54 (9.3)	19 (6.2)	35 (12.8)	
≥4 occasions/week	55 (9.5)	30 (9.8)	25 (9.1)	
**Energy drinks intake**				<0.001 ^M^
Never	444 (76.7)	213 (69.8)	231 (84.3)	
1 occasion/week	62 (10.7)	45 (14.8)	17 (6.2)	
2 to 4 occasions/week	45 (7.8)	29 (9.5)	16 (5.8)	
5 to 6 occasions/week	8 (1.4)	5 (1.6)	3 (1.1)	
1 occasion/day	8 (1.4)	6 (2.0)	2 (0.7)	
2 occasions/day	3 (0.5)	2 (0.7)	1 (0.4)	
3 occasions/day	1 (0.2)	0 (0.0)	1 (0.4)	
≥4 occasions/day	8 (1.4)	5 (1.6)	3 (1.1)	

Abbreviations: Mdn = median; P25 = 25th percentile; P75 = 75th percentile; ^M^ = Mann–Whitney’s U test.

**Table 6:** Consumption of dietary supplements.

	Total	Brazilian	Portuguese	
	*n* (%)	*n* (%)	*n* (%)	*p*
Ingested DS in the prior 12 months	187 (32.3%)	106 (34.8%)	81 (29.6%)	0.213 ^F^
No. of DS ingested (Mdn; P25, P75)	0 (0, 2)	0 (0, 2)	0 (0, 2)	0.194 ^M^

Abbreviations: Mdn = median; P25 = 25th percentile; P75 = 75th percentile; DS = Dietary supplements; ^F^ = Fisher’s exact test; ^M^ = Mann–Whitney’s U test.

**Table 7:** Reasons for using supplements.

	Total	Brazilian	Portuguese
	*n* (%)	*n* (%)	*n* (%)
Prevent/treat illness or injury	158 (84.5)	102 (96.2)	56 (69.1)
Improve cognitive performance	110 (58.8)	68 (64.2)	50 (61.7)
Gain muscle mass	106 (56.7)	66 (62.3)	40 (49.4)
Increase energy/decrease fatigue	97 (51.9)	60 (56.6)	39 (48.1)
Speed up recovery	93 (49.7)	54 (50.9)	29 (35.8)
Compensate for bad eating habits	48 (25.7)	26 (24.5)	22 (27.2)
Lose weight	32 (17.1)	20 (18.9)	13 (16.0)
Decrease stress	30 (16.0)	19 (17.9)	10 (12.3)
Gain weight	15 (8.0)	7 (6.6)	8 (9.9)

**Table 8:** Information sources about dietary supplements.

	Total	Brazilian	Portuguese
	*n* (%)	*n* (%)	*n* (%)
Nutritionist	79 (42.2)	42 (39.6)	37 (45.7)
Medical doctor	44 (23.5)	27 (25.5)	20 (24.7)
Recommendations from athletes	37 (19.8)	24 (22.6)	16 (19.8)
Personal trainer	36 (19.3)	21 (19.8)	15 (18.5)
Friends	32 (17.1)	21 (19.8)	12 (14.8)
Scientific articles	31 (16.6)	21 (19.8)	10 (12.3)
Other healthcare professionals	27 (14.4)	20 (18.9)	9 (11.1)
Other means of communication	27 (14.4)	15 (14.2)	9 (11.1)
Trainer	24 (12.8)	12 (11.3)	6 (7.4)
Relatives	18 (9.6)	12 (11.3)	6 (7.4)
Recommendations from other esports players	8 (4.3)	7 (6.6)	1 (1.2)
Newspapers/magazines	6 (3.2)	5 (4.7)	1 (1.2)
Television	4 (2.1)	4 (3.8)	0 (0.0)
Personal experience/self-taught	3 (1.6)	3 (2.8)	0 (0.0)

**Table 9:** Relation between PREDIMED total score (dependent variable) and characteristics of Portuguese and Brazilian esports players (Univariate ANOVA).

Corrected Model: *p* < 0.001, eta_p_^2^ = 0.173, Adjusted R^2^ = 0.142					
	*n*	EMM	B	*p*	eta_p_^2^
**Nationality**				<0.001	0.052
Portuguese	274	6.195	1.082	<0.001	0.052
Brazilian	305	5.113	(Ref.)		
**Sex**				0.034	0.008
Male	529	5.326	−0.655	0.034	0.008
Female	50	5.981	(Ref.)		
**Age** (quartiles)				0.502	0.004
18 to 20 years	151	5.559	−0.306	0.268	0.002
21 to 23 years	129	5.463	−0.402	0.143	0.004
24 to 29 years	154	5.728	−0.137	0.573	0.001
≥30 years	145	5.865	(Ref.)		
**Higher education**				0.504	0.001
No	221	5.593	−0.121	0.504	0.001
Yes	358	5.714	(Ref.)		
**No. of hours of esports play/day** (terciles)				0.956	0.000
≤2.5	194	5.673	0.059	0.803	0.000
[2.5; 5.0]	175	5.674	0.060	0.786	0.000
≥5	210	5.614	(Ref.)		
**No. of competitions in the past 12 months** (terciles)				0.054	0.010
0	205	5.476	−0.520	0.042	0.007
1 to 3	177	5.490	−0.506	0.026	0.009
≥4	197	5.996	(Ref.)		
**No. of years playing esports** (terciles)				0.874	0.000
≤5	196	5.612	−0.024	0.915	0.000
[5; 10]	214	5.714	0.079	0.721	0.000
>10	169	5.636	(Ref.)		
**Member of an esports team**				0.793	0.000
No	346	5.681	0.054	0.793	0.000
Yes	233	5.627	(Ref.)		
**Level of professionalization**				0.316	0.004
Amateur	360	5.876	0.404	0.201	0.003
Semi-professional	147	5.613	0.140	0.653	0.000
Professional	72	5.472	(Ref.)		
BMI (terciles)				0.207	0.006
<25.0 kg/m^2^	309	5.725	0.334	0.170	0.003
[25.0; 30.0] kg/m^2^	162	5.845	0.454	0.080	0.005
≥30.0 kg/m^2^	108	5.391			
**Caffeine intake** (terciles)				0.356	0.004
≤48 mg	196	5.633	−0.183	0.398	0.001
[48; 224] mg	197	5.513	−0.303	0.152	0.004
>224 mg	186	5.816	(Ref.)		
**DS intake**				<0.001	0.029
No	392	5.280	−0.748	<0.001	0.029
Yes	187	6.028	(Ref.)		
**IGDS9 total score**	579		−0.042	0.002	0.017

Abbreviations: EEM = Estimated marginal mean; B = Regression coefficient; BMI = Body Mass Index; DS = Dietary Supplement; eta_p_^2^ = Partial eta squared; Ref. = Reference.

## Data Availability

The data that support the findings of this study are available from the corresponding author upon reasonable request.

## Supplementary Materials

The following supporting information can be downloaded at: https://www.mdpi.com/article/10.3390/nu15194200/s1, SM1: Google Form containing the questionnaire, SM2: Excel file containing Univariate ANOVA results (Tables S1 and S2).
