# Supplementary material for: Dietary Habits and Gaming Behaviors of Portuguese and Brazilian Esports Players
Source: Nutrients. 2023 Sep 28;15(19):4200. doi: 10.3390/nu15194200 (PMC10574580; doi:10.3390/nu15194200)
Supplement: Supplementary file 1 [file nutrients-15-04200-s001.zip › SM1 Google forms.pdf]

Caracterização de Hábitos Nutricionais e Estilo de Vida de Jogadores de esports

Este questionário insere-se numa Tese de Doutoramento em Ciências do Consumo Alimentar e Nutrição da Universidade do Porto, e tem como objetivo caracterizar os hábitos nutricionais e o estilo de vida de competidores de esports (desportos eletrónicos).
Asseguramos a total confidencialidade das suas respostas e que os dados obtidos serão usados exclusivamente para fins de investigação científica.
O tempo estimado de preenchimento do questionário é de 5 minutos.
Se tiver alguma dúvida envie um email para up201401106@edu.fcna.up.pt

DECLARAÇÃO DE CONSENTIMENTO INFORMADO
Conforme a lei 67/98 de 26 de outubro (Lei da Proteção de Dados Pessoais) e a "Declaração de Helsínquia" da Associação Médica Mundial (Helsínquia 1964).

- Declaro que li este formulário e que compreendi a informação que nele consta.
- Aceito participar de livre vontade no estudo acima descrito.
- Autorizo a divulgação dos resultados obtidos no meio científico.

Se entender que tudo está em conformidade, e se estiver de acordo com a proposta que lhe é feita, inicie o preenchimento do questionário.

*Obrigatório

Características Socio-Demográficas

1. Data de nascimento *

____

Exemplo: 7 de janeiro de 2019

2. Sexo *

Marcar apenas uma oval.

- ⬭ Masculino
- ⬭ Feminino

3. Peso (kg) *

____

4. Altura (cm) *

____

5. Qual o país em que nasceu? *

Marcar apenas uma oval.

- ( ) Portugal
- ( ) Outra: ______________________________

6. Qual é a sua nacionalidade? *

Marcar apenas uma oval.

- ( ) Português
- ( ) Outra: ______________________________

7. Qual foi o nível de escolaridade mais elevado que completou? *

Marcar apenas uma oval.

- ( ) Ensino básico 1º ciclo (4º ano, antigo ensino primário, 4ª classe)
- ( ) Ensino básico 2º ciclo (6º ano, antigo ciclo preparatório)
- ( ) Ensino básico 3º ciclo (9º ano, antigo 5º ano do liceu)
- ( ) Ensino secundário (12ª ano, antigo 7º ano do liceu)
- ( ) Ensino Pós-secundário Não Superior (ex. curso profissional)
- ( ) Ensino Superior (Bacharelato, Licenciatura, Mestrado, Doutoramento)
- ( ) Sem escolaridade
- ( ) Não sabe/ não responde

8. 6. Como define a sua ocupação profissional ou condição perante o trabalho? *

Marcar tudo o que for aplicável.

- [ ] 1. Estudante
- [ ] 2. Trabalhador-estudante
- [ ] 3. Trabalhador por uma remuneração ou lucro (incluindo trabalho não remunerado num negócio de família ou exploração, estágio de aprendizagem ou remunerado, incluindo ainda trabalhadores que não exercem atualmente devido a licença de maternidade, paternidade, por doença ou em férias)
- [ ] 4. Desempregado (sem emprego no período de referência, disponível para trabalhar e a procura de emprego)
- [ ] 5. Outro (reformado, permanentemente incapacitado, trabalhador doméstico, a cumprir serviço militar obrigatório ou serviço comunitário obrigatório)

Hábitos de prática de jogo de esports

9. Quantas horas costuma jogar esports por dia? (Indique hora:min) *

____

10. Em quantas competições de esports participou nos últimos 12 meses? *

____

11. Há quantos anos e meses joga esports? *

____

12. É membro de alguma equipa de esports? *

Marcar apenas uma oval.

- ( ) Sim
- ( ) Não

13. Encontra-se inscrito(a) em alguma liga ou federação de esports? Se sim, qual? *

____

14. Considera-se um(a) jogador(a) amador(a) ou profissional de esports? *

Marcar apenas uma oval.

- ( ) Amador
- ( ) Semi-profissional
- ( ) Profissional

15. Qual é o eGame que costuma jogar de forma competitiva? *

Marcar tudo o que for aplicável.

- [ ] Counter-Strike: Global Offensive (CS:GO)
- [ ] FIFA 2022
- [ ] League of Legends
- [ ] Gran Turismo
- [ ] Valorant
- [ ] Player Unknown's Battlegrounds (PUBG)
- [ ] Fortnite - Battle Royale
- [ ] Dota 2
- [ ] Overwatch
- [ ] Tom Clancy's Rainbow Six
- [ ] Rocket League
- [ ] World of Warcraft
- [ ] Magic the Gathering
- [ ] Starcraft
- [ ] Street Fighter
- [ ] Arena of Valor
- [ ] Apex Legends
- [ ] Hearthstone
- [ ] Dragon Ball Z Fighters
- [ ] Arena of Value
- [ ] Outra: ____________________

16. Indique o seu Rank no eGame que costuma jogar de forma competitiva.

____

Adesão ao Padrão Alimentar Mediterrânico

A segunda parte deste inquérito pretende classificar o seu nível de adesão ao padrão alimentar mediterrânico através do questionário PREDIMED.

Neste grupo de questões pretende-se avaliar os seus hábitos alimentares. Nenhuma resposta é considerada certa ou errada, por isso selecione a opção que mais de adapta à sua alimentação habitual.

Responda às seguintes questões de acordo com aquilo que é a sua alimentação habitual nos 7 dias da semana, indicando o número de vezes (por dia ou por semana) que consome determinados alimentos:

17. **1. Usa o azeite como principal gordura para cozinhar**? *

Marcar apenas uma oval por linha.

	Não	Sim
.	◯	◯

18. **2. Que quantidade de azeite consome num dia**? (incluindo uso para fritar, temperar saladas, refeições fora de casa, etc) *

Marcar apenas uma oval por linha.

	1 ou menos	1	2	3	4 ou mais
**(número de colheres de sopa por dia)**	◯	◯	◯	◯	◯

19. **3. Quantas porções de produtos hortícolas (vegetais, legumes, hortaliças) consome por dia**? (1 porção corresponde a 200 g; considere os hortaliças de acompanhamento como metade de 1 porção e a sopa como 1 porção) *

Marcar apenas uma oval por linha.

	1 ou menos	1	2	3	4 ou mais
**(número de porções por dia)**	◯	◯	◯	◯	◯

20. **4. Quantas peças de fruta (incluindo sumos de fruta natural) consome por dia?** *

Marcar apenas uma oval por linha.

	1 ou menos	1	2	3	4 ou mais
**(número de peças por dia)**	⬭	⬭	⬭	⬭	⬭

21. **5. Quantas porções de carne vermelha, hambúrguer ou produtos cárneos (presunto, salsicha, etc.) consome por dia?** (Considere 1 porção de carne o equivalente à palma da mão = 100-150 g. 1 porção equivale a 3 fatias de fiambre ou 2 fatias de presunto ou 5 salsichas ou 1 hambúrguer. Considere tambémos hambúrgueres de frango/peru e o fiambre de frango ou peru) *

Marcar apenas uma oval por linha.

	1 ou menos	1	2	3	4 ou mais
**(número de porções por dia)**	⬭	⬭	⬭	⬭	⬭

22. **6. Quantas porções de manteiga, margarina, ou natas consome por dia?** (1 porção corresponde a 2 colheres de sobremesa = 12g) *

Marcar apenas uma oval por linha.

	1 ou menos	1	2	3	4 ou mais
**(número de porções por dia)**	⬭	⬭	⬭	⬭	⬭

23. **7. Quantas bebidas açucaradas ou gaseificadas bebe por dia**? (Não considere as bebidas alcoólicas, como a cerveja, nem outras bebidas açucaradas como o leite achocolatado) *

Marcar apenas uma oval por linha.

	1 ou menos	1	2	3	4 ou mais
**(número de porções por dia)**	⬭	⬭	⬭	⬭	⬭

24. **8. Quantos copos de vinho bebe por semana**? (Considere um copo de 100ml) *

Marcar apenas uma oval por linha.

	1 ou menos	1	2	3	4	5	6	7 ou mais
**(número de copos por semana)**	⬭	⬭	⬭	⬭	⬭	⬭	⬭	⬭

25. **9. Quantas porções de leguminosas consome por semana**? (1 porção equivale a 6 colheres de sopa ou 2 colheres de servir ou 1 concha = 150g) *

Marcar apenas uma oval por linha.

	1 ou menos	1	2	3	4	5	6	7 ou mais
**(número de porções por semana)**	⬭	⬭	⬭	⬭	⬭	⬭	⬭	⬭

26. **10. Quantas porções de peixe ou marisco consome por semana**? (Considere 1 porção de peixe o equivalente a uma posta do tamanho da palma da mão, o que corresponde a 100-150g de peixe ou 4-5 unidades ou 200g marisco) *

Marcar apenas uma oval por linha.

	1 ou menos	1	2	3	4	5	6	7 ou mais
**(número de porções por semana)**	⬭	⬭	⬭	⬭	⬭	⬭	⬭	⬭

27. **11. Quantas vezes por semana consome produtos de pastelaria ou doces comerciais (não caseiros), como bolos, bolachas, biscoitos**? (Deve considerar os gelados e todas as bolachas, incluindo bolachas Maria, bolachas de água e sal e bolachas cream-cracker. Os cereais de pequeno-almoço não devem ser considerados) *

Marcar apenas uma oval por linha.

	1 ou menos	1	2	3	4	5	6	7 ou mais
**(número de vezes por semana)**	⬭	⬭	⬭	⬭	⬭	⬭	⬭	⬭

28. **12. Quantas porções (de 30g cada) de oleaginosas (nozes, amêndoas, incluindo amendoins) consome por semana**? (1 porção equivale aproximadamente um punhado de frutos oleaginosos= 30g) *

Marcar apenas uma oval por linha.

	1 ou menos	1	2	3	4	5	6	7 ou mais
**(número de porções por semana)**	⬭	⬭	⬭	⬭	⬭	⬭	⬭	⬭

29. **13. Consome preferencialmente frango, peru ou coelho em vez de vaca, porco, hambúrguer ou salsicha?** *

Marcar apenas uma oval por linha.

	Não	Sim
.	⬭	⬭

30. **14. Quantas vezes por semana consome hortícolas, massa, arroz ou outros pratos confecionados com um refogado (molho à base de tomate, cebola, alho-francês ou alho e azeite)?** *

Marcar apenas uma oval por linha.

	1 ou menos	1	2	3	4	5	6	7 ou mais
**(número de vezes por semana)**	⬭	⬭	⬭	⬭	⬭	⬭	⬭	⬭

31. **15. Com que frequência ingere alimentos tipo fast-food?** *

Marcar apenas uma oval por linha.

	Nunca ou <1 semana	1 por semana	2-4 por semana	5-6 por semana	1 por dia	2 por dia	3 por dia	4 ou mais por dia
**(pizza, hamburguer, etc)**	⬭	⬭	⬭	⬭	⬭	⬭	⬭	⬭

32. **16. Quantos dias por semana toma o pequeno-almoço?** *

Marcar apenas uma oval por linha.

	1 ou menos	1	2	3	4	5	6	7
**(número de vezes por semana)**	⬭	⬭	⬭	⬭	⬭	⬭	⬭	⬭

33. **17. Com que frequência ingere as seguintes bebidas contendo cafeína?** *

Marcar apenas uma oval por linha.

	Nunca ou <1 semana	1 por semana	2-4 por semana	5-6 por semana	1 por dia	2 por dia	3 por dia	4 ou mais por dia
**Café (1 chávena)**	⬭	⬭	⬭	⬭	⬭	⬭	⬭	⬭
**Chá verde (1 chávena)**	⬭	⬭	⬭	⬭	⬭	⬭	⬭	⬭
**Chá preto (1 chávena)**	⬭	⬭	⬭	⬭	⬭	⬭	⬭	⬭
**Coca-cola (1 lata, 1 garrafa, ou 1 copo, 330 ml)**	⬭	⬭	⬭	⬭	⬭	⬭	⬭	⬭
**Bebidas energéticas tipo Red Bull (1 lata, 250 ml)**	⬭	⬭	⬭	⬭	⬭	⬭	⬭	⬭
**Bebida energética tipo Monster (1 lata, 500 ml)**	⬭	⬭	⬭	⬭	⬭	⬭	⬭	⬭

Suplementos Alimentares

Esta seção pretende caracterizar o consumo de suplementos alimentares de competidores de eSports.

34. Ingeriu algum suplemento alimentar nos últimos 12 meses? *

Marcar apenas uma oval.

- Sim
- Não *Avançar para a pergunta 39*

Suplementos Alimentares

Esta seção pretende caracterizar o consumo de suplementos alimentares de competidores de eSports. Nas questões desta seção pode selecionar mais do que uma opção.

35. Indique os tipos de suplementos alimentares que ingeriu nos últimos 12 meses. *

Marcar tudo o que for aplicável.

- [ ] Proteína Whey
- [ ] Caseína
- [ ] Proteína vegetal (soja, ervilha, arroz)
- [ ] Hidratos de carbono (maltodextrina, dextrose/glicose, isomaltulose)
- [ ] Barras desportivas
- [ ] Bebidas desportivas
- [ ] Bebidas energéticas
- [ ] Cafeína
- [ ] Creatina
- [ ] Aminoácidos Essenciais
- [ ] BCAAs (Aminoácidos ramificados)
- [ ] Beta-alanina
- [ ] Tirosina
- [ ] Glutamina
- [ ] Ómega 3
- [ ] Multivitamínico-mineral
- [ ] Complexo multimineral
- [ ] Complexo multivitamínico
- [ ] Vitaminas do complexo B
- [ ] Vitamina B1 (tiamina)
- [ ] Vitamina B2 (riboflavina)
- [ ] Vitamina B3 (niacina)
- [ ] Vitamina B5 (ácido pantoténico)
- [ ] Vitamina B6 (piridoxina)
- [ ] Vitamina B7 (biotina)
- [ ] Vitamina B9 (folato)
- [ ] Vitamina B12 (cobalamina)
- [ ] Vitamina C (ácido ascórbico)
- [ ] Vitamina D3 (colecalciferol)
- [ ] Vitamina E
- [ ] Magnésio
- [ ] Ferro
- [ ] Cálcio
- [ ] Potássio
- [ ] Nitratos / Sumo de beterraba
- [ ] Gingko biloba
- [ ] Ginseng
- [ ] Guaraná
- [ ] Rhodiola rosea
- [ ] Sage (mentol)
- [ ] Flavonóides

- [ ] L-carnitina
- [ ] Glucosamina
- [ ] Condroitina
- [ ] Iodo
- [ ] Levedura de cerveja
- [ ] Outra: ______________________________

36. Porque razões usou o/s suplemento/s? *

Marcar tudo o que for aplicável.

- [ ] Melhorar o desempenho cognitivo
- [ ] Aumentar a concentração/foco
- [ ] Aumentar a energia/diminuir a fadiga
- [ ] Aumentar o controlo emocional
- [ ] Estimulação cognitiva
- [ ] Ajudar a relaxar
- [ ] Diminuir o stress
- [ ] Ganhar massa muscular
- [ ] Ganhar força
- [ ] Melhorar o rendimento desportivo
- [ ] Tornar-me mais rápido
- [ ] Acelerar a recuperação
- [ ] Manter-me saudável
- [ ] Prevenir/tratar doenças ou lesões
- [ ] Aumentar a resistência
- [ ] Corrigir erros alimentares
- [ ] Perder peso
- [ ] Outra: ______________________________

37. Que fontes de informação o influenciaram a adquirir o/s suplemento/s?

Marcar tudo o que for aplicável.

- [ ] Nutricionista
- [ ] Médico
- [ ] Fisioterapeuta
- [ ] Enfermeiro
- [ ] Outro profissional de saúde
- [ ] Treinador
- [ ] Personal trainer
- [ ] Recomendação de Atletas
- [ ] Recomendações de outros jogadores de esports
- [ ] Amigos
- [ ] Familiares
- [ ] Jornais/revistas
- [ ] TV
- [ ] Artigos científicos
- [ ] Outros meios de comunicação
- [ ] Outra: ____________________

38. Onde obteve o/s suplemento/s? *

Marcar tudo o que for aplicável.

- [ ] Loja de suplementos / Ervanária
- [ ] Ginásio
- [ ] Loja de desporto
- [ ] Farmácia
- [ ] Parafarmácia
- [ ] Supermercado
- [ ] Loja online
- [ ] Patrocinador
- [ ] Outra: ____________________

Grau de Dependêndia de Jogos

A última parte deste inquérito pretende caracterizar a possibilidade de "desordem de jogo" ou "vício em jogar" através do questionário Portuguese Internet Gaming Disorder Scale–Short-Form (IGDS9-SF),

Instruções: As questões que se seguem remetem para toda e qualquer atividade relacionada ao tempo despendido em **videojogos** nos últimos 12 meses. As atividades relacionadas com os **videojogos** referem-se, mais concretamente, a qualquer tempo despendido a jogar (quer pela

Internet ou não), jogos em consolas, computadores, portáteis ou em qualquer outro tipo de dispositivo móvel (por exemplo: telemóvel, tablet, etc.).

39. 1. Sente-se preocupado(a) com o seu comportamento em relação aos **videojogos**? (Exemplos: Pensa sobre sessões de jogos anteriores ou antecipa a próxima sessão que irá jogar; Jogar tornou-se a principal atividade do seu dia-a-dia.) *

Marcar apenas uma oval por linha.

	Nunca	Raramente	Algumas vezes	Frequentemente	Quase sempre
.	⬭	⬭	⬭	⬭	⬭

40. 2. Sente-se mais irritado(a), ansioso(a) ou até mesmo triste quando tenta reduzir ou parar de jogar? *

Marcar apenas uma oval por linha.

	Nunca	Raramente	Algumas vezes	Frequentemente	Quase sempre
.	⬭	⬭	⬭	⬭	⬭

41. 3. Sente necessidade de passar cada vez mais tempo a jogar para obter satisfação ou prazer? *

Marcar apenas uma oval por linha.

	Nunca	Raramente	Algumas vezes	Frequentemente	Quase sempre
.	⬭	⬭	⬭	⬭	⬭

42. 4. Falha sistematicamente quando tenta reduzir ou deixar de jogar? *

Marcar apenas uma oval por linha.

	Nunca	Raramente	Algumas vezes	Frequentemente	Quase sempre
.	⬭	⬭	⬭	⬭	⬭

43. 5. Perdeu o interesse por outras atividades de lazer em resultado do seu envolvimento com os **videojogos**? *

Marcar apenas uma oval por linha.

	Nunca	Raramente	Algumas vezes	Frequentemente	Quase sempre
.	⬭	⬭	⬭	⬭	⬭

44. 6. Já continuou a jogar com a mesma intensidade mesmo a saber que isso estava a causar problemas entre si e outras pessoas? *

Marcar apenas uma oval por linha.

	Nunca	Raramente	Algumas vezes	Frequentemente	Quase sempre
.	⬭	⬭	⬭	⬭	⬭

45. 7. Já escondeu de algum familiar, terapeuta ou outra pessoa, a quantidade de tempo que passou a jogar? *

Marcar apenas uma oval por linha.

	Nunca	Raramente	Algumas vezes	Frequentemente	Quase sempre
.	⬭	⬭	⬭	⬭	⬭

46. 8. Joga para evitar ou aliviar sentimentos difíceis (Exemplos: desamparo, culpa, ansiedade)? *

Marcar apenas uma oval por linha.

	Nunca	Raramente	Algumas vezes	Frequentemente	Quase sempre
.	⬭	⬭	⬭	⬭	⬭

47. 9. Já colocou em risco, perdeu um relacionamento importante, oportunidade de trabalho ou educacional, por causa dos **videojogos**? *

Marcar apenas uma oval por linha.

	Nunca	Raramente	Algumas vezes	Frequentemente	Quase sempre
.					

Este conteúdo não foi criado nem aprovado pela Google.

Google Formulários
